# Analysis of the association between dietary patterns and nonalcoholic fatty liver disease in a county in Guangxi

**DOI:** 10.1186/s12876-023-02864-7

**Published:** 2023-09-13

**Authors:** Song Xiao, Ziqi Chen, Tingyu Mai, Jiansheng Cai, Yulu Chen, Xu Tang, Ruoyu Gou, Tingyu Luo, Kailian He, Tingjun Li, Jian Qin, Zhiyong Zhang, You Li

**Affiliations:** 1 Department of Environmental Health and Occupational Medicine, School of Public Health, Guilin Medical University, No. 1 Zhiyuan Road, Lingui District, Guilin, Guangxi 541199 China; 2 Guangxi Health Commission Key Laboratory of Entire Lifecycle Health and Care, Guilin Medical University, No. 1 Zhiyuan Road, Lingui District, Guilin, Guangxi 541199 China; 3 Guangxi Key Laboratory of Tumor Immunology and Microenvironmental Regulation, Guilin Medical University, No. 1 Zhiyuan Road, Lingui District, Guilin, Guangxi 541199 China; 4 Department of Environmental and Occupational Health, School of Public Health, Guangxi Medical University, Shuangyong Road No.22, Nanning, Guangxi province 530021 PR China

**Keywords:** Nonalcoholic fatty liver disease, Dietary pattern, Diet, Liver

## Abstract

**Background:**

This study aims to investigate the relationship between different dietary patterns and non-alcoholic fatty liver disease (NAFLD).

**Methods:**

Residents over 30 years old in the ecological longevity cohort in Gongcheng Yao Autonomous County, Guangxi Province were the research objects selected from 2018 to 2019. Physical examination, baseline population survey, and food frequency questionnaire (FFQ) survey were conducted. Dietary patterns were analyzed by factor analysis. Influencing factors of NAFLD were analyzed by multiple logistic regression.

**Results:**

NAFLD was diagnosed in 241 of 2664 participants based on ultrasonography, and the detection rate was 9.0%. Factor analysis yielded a total of three dietary patterns, namely, traditional Chinese, Western, and cereal-potato dietary patterns. Results of multivariate logistic regression analysis showed that after adjusting for confounding factors, participants in the highest quartile of the Western dietary pattern exhibited a higher prevalence of NAFLD (OR = 2.799; 95% CI: 1.620–4.837; *p* < 0.05) than participants in the lowest quartile. Participants in the highest quartile of the cereal-potato pattern exhibited a decreased risk of NAFLD compared with those in the lowest quartile (OR = 0.581; 95% CI: 0.371–0.910, *p* < 0.05). The traditional Chinese patterns did not show any association with the risk of NAFLD.

**Conclusions:**

The Western dietary pattern increases the risk of NAFLD, whereas the cereal-potato dietary pattern reduces the risk of NAFLD. It is important for the prevention and control of NAFLD to adhere to the cereal-potato dietary.

## Background

Nonalcoholic fatty liver disease (NAFLD) is a clinicopathological syndrome characterized by steatosis and fat deposition in hepatic parenchymal cells with no history of excessive alcohol consumption [1]. With the improvement of living standards, the prevalence of NAFLD has increased year by year to more than 25% worldwide [2], and the prevalence among adults in Western countries ranges from 20 to 33% [3]. This was 20.1% among Chinese adults [4]. NAFLD increases the risk of developing metabolic syndrome and cardiovascular disease [5, 6], and if not detected, diagnosed, and treated early, it may develop into advanced fibrosis, cirrhosis, and hepatocellular carcinoma (HCC) [7]. Studies have shown that fatty liver is the result of a combination of genetic and environmental factors, with lifestyle concerns such as nutrition [8–10]. The intake of dietary fat, carbohydrates, and other nutrients is closely related to fat deposition in the liver [11]. No clinically effective drugs are available for the treatment of NAFLD, and the recommended treatment is to change the lifestyle and dietary patterns, such as controlling the intake of certain components and increasing exercise [12–14]. The results of numerous epidemiological studies have shown a strong association between one or more nutrients or foods and NAFLD. Compared with a single food or nutrient, dietary pattern can comprehensively reflect the dietary exposure of a group and predict disease occurrence as a comprehensive indicator of individual dietary nutritional intake [15]. Therefore, it is necessary to investigate the relationship between the occurrence of NAFLD and different dietary patterns. The residents of Gongcheng Yao Autonomous County, Guangxi are mostly from ethnic minorities, and the area is the “hometown of longevity in China,” with more long-lived elderly people and lower incidence of metabolic syndrome and cardiovascular metabolic diseases compared with northern areas [16]. To understand the prevalence of NAFLD in this minority region and its relationship with dietary patterns can provide a theoretical basis for the prevention and control of NAFLD and for reasonable dietary guidance in this region.

## Method

### Research objects

**Fig. 1:**
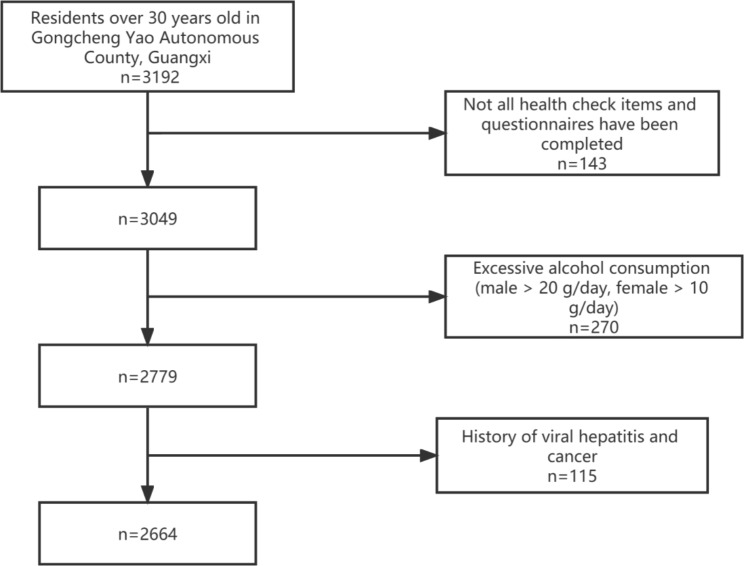
Flowchart of the study participants

From December 2018 to November 2019, the ecological longevity cohort of residents over 30 years old in Gongcheng Yao Autonomous County, Guangxi Province was selected to conduct physical examination, baseline population survey, and food frequency questionnaire (FFQ) survey. The inclusion criteria were as follows: (1) permanent residents of Gongcheng Yao Autonomous County, Guangxi Zhuang Autonomous Region; (2) over 30 years old; and (3) be able to cooperate with the completion of all health examination items and questionnaire survey. We then excluded subjects who had not completed a physical examination and questionnaire, had a history of viral hepatitis and cancer, and drank excessively (men > 20 g/ day, women > 10 g/ day).The final study included 2,664 subjects (1,016 men and 1,648 women) (Fig. 1).

### Demographic characteristics

A self-designed questionnaire was used to conduct an interview survey by trained and qualified investigators. The contents included demographic characteristics (gender, age, education level, marital status, occupation, and others), lifestyle habits (smoking, drinking, physical activity, and others), and medical history.

### Clinical assessments

During the measurement, participants were barefoot and did not wear a hat. Weight (kg) was measured by an electronic digital scale. Height (m) was measured by a safe portable height meter, which was accurate to one decimal place. Body mass index (BMI) = weight (kg)/height^2^ (m^2^) was calculated according to the formula. Participants were classified as underweight (BMI < 18.5 kg/m^2^), normal weight (BMI 18.5–23.9 kg/m^2^), overweight (BMI 24.0–27.9 kg/m^2^), and obese (BMI ≥ 28.0 kg/m^2^) [17]. When the participant was breathing smoothly, the waist circumference (WC) at the midpoint between the 12 lower costal margins and the upper iliac crest was measured by a uniformly trained medical examiner who used an inelastic tape measure accurate to 1 mm. Hip circumference is measured by circling the ruler at the highest point of the hip. The waist-to-hip ratio (WHR) was calculated as waist circumference (cm) divided by hip circumference (cm). Systolic and diastolic blood pressure were measured twice, and average values were recorded. Hypertension refers to systolic blood pressure (SBP) ≥ 140 mmHg (1 mmHg = 0.133 kPa) or diastolic blood pressure (DBP) ≥ 90 mmHg or previously diagnosed hypertension. Diabetes mellitus refers to FBG ≥ 7.0 mmol/L or HbA1c ≥ 6.5% or previously diagnosed diabetes mellitus (all measuring instruments are strictly calibrated before use).Physical activity levels were measured in minutes per week, with adequate physical activity being defined as ≥ 150 min per week and inadequate as less than 150 min per week [18].

### Laboratory tests

After fasting for 8–12 h, participants’ venous blood was collected by a nurse at the local township health center physical examination center. Subsequently, fasting blood glucose (FBG), serum total cholesterol (TC), triglycerides (TG), high-density lipoprotein cholesterol (HDL-C), and LDL-C, fasting blood glucose (FPG), glycated hemoglobin (HbA1C), glutathione aminotransferase (AST), alanine aminotransferase (ALT), uric acid (UA), and others were determined.

### Dietary intake and extraction of dietary patterns

Food intake was obtained by using an FFQ [19] to obtain the frequency of intake of various foods, mainly 109 foods that are frequently consumed by Chinese people, and the intake amount of each food by the survey respondents in the last year. For each food, respondents were asked about the frequency of intake (never, 1–3 times per month, 1–2 times per week, 3–4 times per week, 5–6 times per week, 1 time per day, 2 times per day, and 3 times per day) and the amount of each intake. Finally, the average daily intake of each food group was calculated for each person based on the intake and consumption frequency of each food group. The Kaiser-Meyer-Olkin (KMO) statistic and Bartlett’s spherical test were used to determine whether the conditions for factor analysis were met. The number of retained common factors was determined by using the gravel plot and the characteristic root > 1 as the inclusion criteria. The food type of each factor was represented by the components with a factor loading of > 0.3, or the dietary pattern was classified and named according to the larger factor loadings, i.e., the relevance of the food type to the diet and the food characteristics after rotation. Each dietary pattern was classified into four levels (Q1, Q2, Q3, and Q4) according to the quartiles of dietary factor scores. Q1 indicated the least inclination to this dietary pattern, and Q4 indicated the most inclination to this dietary pattern [20].

### Definition of non-alcoholic fatty liver disease

NAFLD was defined as moderate to severe hepatic steatosis found on ultrasound, no excessive alcohol consumption (> 20 g daily alcohol intake for men and > 10 g for women), no use of fat-forming drugs in the past 6 months, no exposure to hepatotoxins, and no history of bariatric surgery [21].

### Statistical analysis

Count data expressed as a percentage or forming ratio, the normal distribution of measurement data to $\stackrel{-}{\text{x}}$±s. χ^2^ test, Mann–Whitney U test or ANOVA were used for comparison among the groups. Principal component analysis was used to analyze dietary patterns, and multiple logistic regression was used to analyze and adjust confounding factors, such as age, gender, smoking, alcohol consumption, blood pressure, and blood lipid, to explore the relationship between each dietary pattern and the risk of NAFLD. EpiData 3.1 was adopted to establish a database for unified data entry. All statistical analyses were performed using SPSS 28.0, and a *p* value of < 0.05 was statistically significant.

## Results

### Dietary patterns

The dimension reduction method of factor analysis was used to conduct principal component analysis and the classification of dietary types. The KMO test statistic was 0.845, and Bartlett spherical test was *p* < 0.001; it was suitable for factor analysis. The number of common factors, i.e., the number of dietary patterns, was determined according to the criteria of the lithorubble map and the characteristic root > 1. Three dietary patterns were extracted, the characteristic roots were 2.703, 1.576, and 8.770 respectively, and the variance contribution rates were 22.52%, 13.12%, and 8.77%, respectively. After the factor component matrix was rotated by the method of maximum variance orthogonal rotation, food categories with a factor load of > 0.50 had a strong relationship with the dietary pattern, and the dietary pattern was named according to the factor load and food component characteristics. Factor 1 was the traditional Chinese model, which mainly comprised vegetables, fruits, bean products, nuts, mushrooms, eggs, and milk. Factor 2 was the Western model, which was characterized by high intake of red meat, processed meat, offal, white meat, fish, seafood, and alcoholic beverages. Factor 3 was the cereal–potato pattern, and the main components were whole grains and tubers. The factor load matrix of these dietary patterns is shown in Table 1.

**Table 1:** Factor loadings of the three dietary patterns of 2664 study participants

Food groups(% variance)	Dietary patterns		
	Traditional Chinese(22.52%)	Western(13.12%)	Cereal-potato(8.77%)
Cereal and potato	0.323	—	0.582^†^
Vegetables	0.627^†^	—	—
Fruits	0.668^†^	0.245	—
Bean products	0.69^†^	0.218	—
Nuts	0.618^†^		—
Preserved, red meat & offal	0.225	0.520^†^	—
White meat	0.251	0.561^†^	—
Aquatic products	—	0.668^†^	0.212
Egg and milk	0.539^†^	—	—
Mushrooms	0.617^†^	—	—
Alcoholic drinks	—	0.609^†^	—
Oil and salt	0.248	—	—

Absolute values < 0.2 were excluded for simplicity

†Food groups with factor load ≥ 0.50.

### Participant characteristics

A total of 2664 subjects were included, as follows: 1648 females (mean age: 56.6 ± 11.1 years old) and 1016 males (mean age: 58.6 ± 12.4 years old). A total of 241 patients with NAFLD were detected with a detection rate of 9.0%. There were significant differences in age, smoking, alcohol consumption, and overweight/obesity between NAFLD patients and non-patients (*p* < 0.05). There was no significant difference in gender, marital status, education level, physical activity, and occupation between participants with and without NAFLD (*p* > 0.05), as shown in Table 2.

**Table 2:** Comparison of demographic characteristics between participants with and without NAFLD

Characteristics	Total, n	NAFLD		p
		Yes	No	
Demographic	2664	241(9.0)	2423(91.0)	
Age group, n (%)				< 0.001
30-59years	1302	142(58.9)	1160(47.9)	
60-94years	1362	99(41.1)	1263(52.1)	
Sex				0.685
Female	1648	152(63.1)	1496(61.7)	
Male	1016	89(36.9)	927(38.3)	
Ethnicity, n (%)	2664			0.392
Han	548	46(19.1)	502(20.7)	
Yao	1987	187(77.6)	1800(74.3)	
Others	129	8(3.3)	121(5.0)	
Marital status, n (%)				0.239
Unmarried or divorced	436	33(13.7)	403(16.6)	
Married or cohabiting	2228	208(86.3)	2020(83.4)	
Educational Level, n (%)				0.553
Incomplete primary or lower	1109	96(39.8)	1013(41.8)	
Completed primary or higher	1555	145(60.2)	1410(58.2)	
Physical activity				0.286
Inadequate	665	67(27.8)	598(24.7)	
Adequate	1999	174(72.2)	1825(75.3)	
Smoking, n (%)				0.003
No	2177	214(88.8)	1963(81.0)	
Yes	487	27(11.2)	460(19.0)	
Drinking, n (%)				
No	1781	236(97.9)	1545(63.8)	< 0.001
Yes	883	5(2.1)	878(36.2)	
Occupational, n (%)				0.415
Farmers	2436	217(90.0)	2219(91.6)	
Else	228	24(10.0)	204(8.4)	
BMI (kg/m^2^)	2664	26.6 ± 3.1	22.4 ± 3.2	< 0.001
WC, cm	2664	89.1 ± 9.1	77.2 ± 9.7	< 0.001
Overweight/Obese (%)	699	165(68.5)	534(22.0)	< 0.001
Hypertension, n (%)	1113	127(52.7)	986(40.7)	< 0.001
Diabetes, n (%)	318	54(22.4)	264(10.9)	< 0.001

Continuous variables are presented as Mean ± SD.

*P values for continuous variables (t test) and for categorical variables (chi-square test).

The BMI, systolic and diastolic blood pressure, WC, fasting blood glucose, HbA1c levels, ALT, total cholesterol, low-density lipoprotein cholesterol, triglycerides, and uric acid in the NAFLD group were higher than those in the non-NAFLD group, and the differences were all statistically significant (*p* < 0.05). No significant difference was found in AST and total energy intake between participants with and without NAFLD (*p* > 0.05). (Table 3)

**Table 3:** Comparison of the main phenotypic characteristics between participants with and without NAFLD

Variables	Total, n	NAFLD		*p*
		Yes	No	
BMI (kg/m^2^)	2664	26.6 ± 3.1	22.4 ± 3.2	< 0.001
WC (cm)	2664	89.1 ± 9.1	77.2 ± 9.7	< 0.001
SBP (mmHg)	2664	139.4 ± 24.9	135 ± 23.9	0.007
DBP (mmHg)	2664	86.5 ± 16.2	81.8 ± 14.4	< 0.001
FBG (mmol/L)	2664	5.3 ± 1.7	4.9 ± 1.2	0.001
HbA1C (%)	2664	6.2 ± 1.2	5.8 ± 0.8	< 0.001
AST (IU/L)	2664	23 ± 8.3	23 ± 8.2	0.955
ALT (IU/L)	2664	25.3 ± 12.4	19.1 ± 9.4	< 0.001
TC (mmol/L)	2664	5.7 ± 1.0	5.5 ± 1.1	0.009
HDL-C (mmol/L)	2664	1.5 ± 0.3	1.8 ± 0.4	< 0.001
LDL-C (mmol/L)	2664	3.7 ± 1.0	3.4 ± 1.0	< 0.001
TG (mmol/L)	2664	2.2 ± 1.4	1.3 ± 1.1	< 0.001
UA (mmol/L)	2664	354.7 ± 89.6	300.4 ± 92.1	< 0.001
Total energy intake (kcal)	2664	1563.9 ± 883.0	1624.3 ± 854.0	0.296

Continuous variables are presented as Mean ± SD.

### Dietary pattern and NAFLD

Table 4 showed the demographic characteristics of the study participants across the quartile categories of the dietary pattern scores. Participants in the highest quartile of the traditional Chinese dietary pattern were more likely to be female, non-smokers, and non-drinkers. They had plenty of physical exercise and a lower prevalence of hypertension and diabetes. They had lower WHR, SBP, DBP, FPG, HbA1C, AST, TC, HDL-C, TG, and UA values than those in the lowest quartile. Compared with the lowest quartile of the Western pattern dietary pattern, those participants in the highest quartile were more likely to be male, younger, smokers, and drinkers. They exhibited higher BMI, WC, AST, ALT, and UA values. Participants in the highest quintile of the cereal-potato pattern were more likely to be older, non-smokers, and non-drinkers and exhibited significantly higher systolic and diastolic blood pressure. They had a higher prevalence of hypertension and NAFLD than those in the lowest quartile.

**Table 4:** Characteristics of the study participants by quartile (Q) categories of dietary pattern scores

	Traditional Chinese dietary pattern		P	Western dietary pattern		P	Cereal-potato dietary pattern		P
	Q1 (n = 666)	Q4 (n = 666)		Q1 (n = 666)	Q4 (n = 666)		Q1 (n = 666)	Q4 (n = 666)	
Sex(%)			0.001			< .001			0.221
Female	363(54.5)	424(63.7)		545(81.8)	228(34.2)		381(57.2)	403(60.5)	
Male	303(45.5)	242(36.3)		121(18.2)	438(65.8)		285(42.8)	263(39.5)	
Age, y	62.12 ± 11.51	55.08 ± 12.21	< .001	59.44 ± 12.45	57.38 ± 12.02	0.002	56.85 ± 11.763	58.84 ± 12.101	0.02
Ethnicity,n (%)			0.423			0.708			0.916
Han	21(17.4)	253(20.9)		26(21.8)	251(20.7)		21(19.1)	251(20.5)	
Yao	95(78.5)	886(73.2)		89(74.8)	901(74.3)		84(76.4)	911(74.5)	
Others	5(4.1)	72(5.9)		4(3.4)	61(5.0)		5(4.5)	60(4.9)	
Marital status, n (%)			< .001			< .001			0.234
Unmarried or divorced	158(23.7)	77(11.6)		134(20.1)	84(12.6)		116(17.4)	100(15)	
Married or cohabiting	508(76.3)	589(88.4)		532(79.9)	582(87.4)		550(82.6)	566(85)	
Educational Level (n, %)			0.936			0.636			0.636
Incomplete primary or lower	52(43.0)	525(43.4)		53(44.5)	513(42.3)		53(44.5)	513(42.3)	
Completed primary or higher	69(57.0)	686(56.6)		66(55.5)	700(57.7)		66(55.5)	700(57.7)	
Physical activity			< .001			0.492			0.041
Inadequate	206(30.9)	149(22.4)		179(26.9)	168(25.2)		178(26.7)	146(21.9)	
Adequate	460(69.1)	517(77.6)		487(73.1)	498(74.8)		488(73.3)	520(78.1)	
Smoking, n (%)			< .001			< .001			0.01
No	498(74.8)	557(83.6)		621(93.2)	429(64.4)		521(78.2)	558(83.8)	
Yes	168(25.2)	109(16.4)		45(6.8)	237(35.6)		145(21.8)	108(16.2)	
Drinking, n (%)			< .001			< .001			0.002
No	390(58.6)	462(69.4)		583(87.5)	256(38.4)		404(60.7)	457(68.6)	
Yes	276(41.4)	204(306)		83(12.5)	410(61.6)		262(39.3)	209(31.4)	
Occupational, n (%)			< .001			< .001			0.212
Farmers	630(94.6)	575(86.3)		627(94.1)	580(87.1)		613(92)	600(90.1)	
Else	36(5.4)	91(13.7)		39(5.9)	86(12.9)		53(8)	66(9.9)	
Overweight/Obese (%)	167(25.1)	184(27.6)	0.29	176(26.4)	188(28.2)	0.461	177(26.6)	191(28.7)	0.391
NAFLD(%)	55(8.3)	66(9.9)	0.294	76(11.4)	43(6.5)	0.02	44(6.6)	66(9.9)	0.029
Hypertension (%)	324(48.6)	245(36.8)	< .001	295(44.3)	272(40.8)	0.202	242(36.3)	289(43.4)	0.009
Diabetes (%)	113(17.0)	64(9.6)	< .001	74(11.1)	94(14.1)	0.099	78(11.7)	79(11.9)	0.932
BMI (kg/m^2^)	22.66 ± 3.37	22.98 ± 3.307	0.081	22.65 ± 3.454	23.04 ± 3.373	0.036	22.73 ± 3.419	22.95 ± 3.418	0.239
WC (cm)	78.21 ± 10.67	78.91 ± 9.82	0.211	77.84 ± 9.78	79.29 ± 9.59	0.007	78.83 ± 10.21	78.52 ± 10.23	0.586
WHR	0.89 ± 0.08	0.87 ± 0.09	< .001	0.88 ± 0.08	0.88 ± 0.087	0.845	0.88 ± 0.09	0.873 ± 0.08	0.147
SBP (mmHg)	139.8 ± 24.87	132.94 ± 23.35	< .001	136.81 ± 23.64	134.44 ± 24.68	0.074	131.81 ± 21.39	137.03 ± 25.05	< .001
DBP (mmHg)	83.52 ± 14.66	81.57 ± 14.21	0.014	81.97 ± 14.18	82.29 ± 14.39	0.69	81.03 ± 14.05	82.72 ± 15.26	0.035
FPG (mmol/L)	5.17 ± 1.41	4.86 ± 1.29	< .001	4.99 ± 1.37	5 ± 1.35	0.877	4.91 ± 1.32	4.94 ± 1.25	0.699
HbA1C(%)	5.92 ± 1.01	5.77 ± 0.87	0.004	5.85 ± 0.95	5.83 ± 0.84	0.702	5.78 ± 0.93	5.84 ± 0.83	0.192
AST (IU/L)	24.91 ± 8.85	22.35 ± 7.96	< .001	22.4 ± 6.6	24.45 ± 10.2	< .001	23.25 ± 7.97	22.52 ± 6.72	0.073
ALT (IU/L)	20.87 ± 10.88	19.77 ± 10.2	0.059	19.05 ± 8.84	20.58 ± 10.62	0.004	19.87 ± 10.34	19.69 ± 9.92	0.746
TC (mmol/L)	5.66 ± 1.15	5.43 ± 1.06	< .001	5.5 ± 1.08	5.59 ± 1.05	0.126	5.51 ± 1.04	5.53 ± 1.04	0.642
HDL-C (mmol/L)	1.83 ± 0.45	1.7 ± 0.38	< .001	1.75 ± 0.41	1.79 ± 0.46	0.14	1.76 ± 0.43	1.74 ± 0.41	0.531
LDL-C (mmol/L)	3.46 ± 1.02	3.46 ± 0.98	0.977	3.41 ± 0.99	3.44 ± 0.95	0.558	3.4 ± 0.98	3.47 ± 0.98	0.206
TG (mmol/L)	1.49 ± 1.37	1.35 ± 1.02	0.044	1.37 ± 0.97	1.5 ± 1.59	0.062	1.41 ± 1.31	1.4 ± 1.3	0.888
UA (mmol/L)	319.09 ± 100.56	304.69 ± 88.62	0.006	290.03 ± 88.12	337.51 ± 95.82	< .001	312.85 ± 94.26	305.42 ± 92.52	0.147

Note: According to the factor score quartile, from the lowest to the highest, it was Q1, Q2, Q3 and Q4 (Q4 was the most inclined to this type of diet, Q1 was the least inclined to this type of diet), 666 people in each group

Categorical variables are presented as sum and percentages, and continuous variables are presented as Mean ± SD

Table 5 showed food and nutrient intakes across quartiles (Q) of the dietary patterns. In the quartile of the traditional Chinese dietary pattern, there were significant differences were found in the intake for each of the twelve food groups, and participants in the top quartile of the traditional Chinese dietary pattern had a higher intake of energy, protein, fat, carbohydrate, and saturated fatty acid than those in the lowest quartile. In addition, we did not find a significant difference in the intake of nuts, egg, and milk, and mushrooms intake across quartiles of the Western dietary pattern. Meanwhile, no significant difference was observed in the intake of refined preserved, red meat, and offal, mushrooms, and energy across quartiles of this pattern.

**Table 5:** Food and nutriment intakes across quartiles (Q) of the dietary patterns at baseline in Guangxi Residents

	Traditional Chinese dietary pattern		P	Western dietary pattern		P	Cereal-potato dietary pattern		P
	Q1 (n = 666)	Q4 (n = 666)		Q1 (n = 666)	Q4 (n = 666)		Q1 (n = 666)	Q4 (n = 666)	
Cereals and tubers, g/d	241.53 ± 196.36	542.12 ± 381.23	< .001	351.86 ± 302.44	456.77 ± 372.99	< .001	225.38 ± 185.38	717.63 ± 372.41	< .001
Vegetable, g/d	137.34 ± 101.33	604.57 ± 413.74	< .001	281.01 ± 312.25	440.79 ± 360.31	< .001	288.57 ± 275.83	483.02 ± 367.41	< .001
Fresh fruits, g/d	79.6 ± 87.64	532.44 ± 401.73	< .001	188.13 ± 235.05	357.31 ± 393.98	< .001	216.33 ± 251.38	372.13 ± 386.6	< .001
bean, g/d	8.63 ± 11.18	79.23 ± 77.02	< .001	21.15 ± 34.35	52.67 ± 66.87	< .001	29.23 ± 46.62	47.44 ± 61.02	< .001
Nuts, g/d	3.15 ± 6.24	33.22 ± 43.95	< .001	17.34 ± 36.48	16.64 ± 26.98	0.693	22.25 ± 40.17	11.04 ± 16.55	< .001
Marinating, red meat and offal, g/d	47 ± 70.08	94.47 ± 95.65	< .001	24.12 ± 25.88	133.53 ± 163.53	< .001	83.03 ± 147.01	72.56 ± 81.98	0.109
White meat, g/d	7.72 ± 18.69	19.11 ± 28.06	< .001	3.19 ± 4.77	26.98 ± 42.81	< .001	11.48 ± 23.15	16.71 ± 34.79	0.001
Fish, seafood and aquatic products, g/d	13.17 ± 40.16	25.08 ± 31.65	< .001	3.85 ± 5.95	40.89 ± 53.18	< .001	12.92 ± 19.86	29.7 ± 50.94	< .001
Eggs, milk and yogur, g/d	22.26 ± 27.1	130.64 ± 120.45	< .001	69.15 ± 93.03	75.99 ± 92.89	0.18	79.71 ± 102.21	68.29 ± 72.84	0.019
Mushrooms/fungi, g/d	1.09 ± 2.08	13.89 ± 21.29	< .001	5.89 ± 17.07	5.97 ± 11.9	0.928	5.75 ± 15.89	6.34 ± 11.93	0.439
Alcoholic Beverages, g/d	88.92 ± 217.49	37 ± 91.76	< .001	1.85 ± 10.65	150.95 ± 233.28	< .001	84.26 ± 214.63	35.16 ± 97.24	< .001
Oil and seasoning, g/d	34.25 ± 20.97	60.77 ± 60.66	< .001	37.32 ± 22.58	55.18 ± 53.72	< .001	82.38 ± 58.18	30.54 ± 17.08	< .001
Energy, g/d	1124.13 ± 624.39	2400.15 ± 968.16	< .001	1195.59 ± 655.89	2332.8 ± 1021.3	< .001	1926.59 ± 1021.07	1845.51 ± 821.45	0.111
Protein, g/d	24.62 ± 15.67	73.17 ± 35.04	< .001	31.83 ± 23.4	65.82 ± 39.45	< .001	44.87 ± 34.62	56.01 ± 30.28	< .001
Fat, g/d	46.11 ± 29.75	107.99 ± 66.39	< .001	50.99 ± 33.63	102.53 ± 71.53	< .001	109.56 ± 71.7	62.68 ± 35.35	< .001
Carbohydrate, g/d	119.46 ± 58.48	284.77 ± 121.09	< .001	158.04 ± 92.35	236.64 ± 122.95	< .001	165.26 ± 93.64	260.8 ± 114.23	< .001
Saturated fatty acid, g/d	11.31 ± 8.25	26.25 ± 15.19	< .001	11.8 ± 7.41	26.47 ± 19.68	< .001	25.42 ± 18.6	16.25 ± 10.06	< .001
Monounsaturated fatty acids, g/d	18.41 ± 12.49	39.92 ± 27.2	< .001	19 ± 12.49	39.55 ± 29.59	< .001	43.48 ± 29.65	22.58 ± 13.41	< .001
Polyunsaturated fatty acids, g/d	11.27 ± 6.65	27.58 ± 19.97	< .001	14.43 ± 10.61	23.07 ± 17.599	< .001	29.41 ± 19.42	14.17 ± 7.67	< .001

Data are shown as mean § standard deviation.

Table 6 showed the associations between dietary patterns and the risk of NAFLD. After adjusting for confounding factors, such as age, sex, smoking status, waist circumference, total energy intake, diabetes, and hypertension in Model 1, subjects in the highest quartile of the Western pattern scores had greater prevalence ratios for NAFLD (OR = 2.799; 95% CI: 1.620–4.837; *p* < 0.05) than did those in the lowest quartile, whereas those in the highest quartile of the cereal and potato pattern score had lower OR for NAFLD (OR = 0.581; 95% CI: 0.371–0.910, *p* < 0.05) than did those in the lowest quartile. In the BMI-adjusted model 2, the Western model was still positively associated with NAFLD and the cereal and potato models were negatively associated with NAFLD. However, the traditional Chinese dietary patterns showed no association with the risk of NAFLD.

**Table 6:** Multivariable models adjusted for non-alcohol fatty liver disease across the quartile (Q) categories of the dietary patterns

	Model 1 ^a^	*p* ^*^	Model 2 ^b^	*p* ^*^
	OR (95% CI)		OR (95% CI)	
Traditional Chinese		0.172		0.207
Q1	1 (reference)		1 (reference)	
Q2	0.762(0.497–1.167)		1.345(0.879–2.06)	
Q3	1.233(0.769–1.977)		1.023(0.686–1.525)	
Q4	0.947(0.554–1.62)		1.438(0.942–2.197)	
Western		0.002		0.001
Q1	1 (reference)		0.434(0.28–0.674)	
Q2	1.248(0.843–1.847)		0.488(0.313–0.76)	
Q3	1.773(1.143–2.751)		0.667(0.419–1.06)	
Q4	2.799(1.620–4.837)		0.79(0.51–1.25)	
Cereal-potato		0.028		0.1
Q1	1 (reference)		1 (reference)	
Q2	0.626(0.398–0.985)		1.598(1.034–2.469)	
Q3	0.515(0.327–0.809)		1.053(0.696–1.593)	
Q4	0.581(0.371–0.910)		0.963(0.64–1.45)	

^a^ Adjusted for age, sex, smoking status, waist circumference, total energy intake, diabetes, and hypertension

^b^ Additionally adjusted for BMI

## Discussion

Among the 2664 subjects included in the study, 241 patients with MAFLD were detected, with a detection rate of 9.0%, which is lower than the prevalence of non-alcoholic fatty liver disease in the United States aged 18–59 years (41.5% in men and 29.9% in women, respectively)[22]. This may be due to the differences in region, diet and age. In this study, people over 30 years old, mainly female and ethnic minorities, were selected.

Diet is an important causative factor for NAFLD and a manageable risk factor [23]. The treatment of NAFLD still focuses on dietary and lifestyle interventions, such as adjusting the healthy dietary structure, losing weight, and increasing exercise, due to the lack of effective and safe clinical treatments and drugs for NAFLD. These treatment options are all based on the principle of controlling total energy and sugar intake and reducing the burden on liver cells by reducing liver fat accumulation to prevent and treat NAFLD. The study extracted the data from the questionnaire through factor analysis. Three main dietary patterns were constructed: the traditional Chinese, the Western, and the cereal-potato patterns. Further analysis showed that food consumption in the “Western” dietary pattern was associated with an increased risk of NAFLD. That in the cereal-potato dietary pattern was associated with a decreased risk of NAFLD. The traditional Chinese dietary pattern was not associated with NAFLD. These associations were independent of gender, age, physical activity, BMI, smoking status, and blood pressure.

In our analysis, the traditional Chinese model was characterized by a high consumption of vegetables, fruits, legumes, eggs, milk, and mushrooms. It was not associated with NAFLD risk. Our results are consistent with those of Chao-Qun Yang et al. [24] but different from those of Xiaonan Liu et al. [25]. The report claims that the traditional Chinese model is associated with NAFLD risk reduction. The complexity of dietary patterns may explain the different results from study to study. This type of dietary pattern contains a large amount of legumes. On the one hand, legumes can reduce the level of total cholesterol and are low-energy foods that can improve lipid metabolism and reduce lipid peroxidation. They are negatively correlated with the risk of NAFLD [26–28]. On the other hand, due to their low glycemic index (GI), legumes reduce the rate of glucose absorption in the intestine, thus reducing the risk of NAFLD [29]. In addition, vegetables and fruits in this dietary pattern are rich in dietary fiber. High intake of dietary fiber is significantly negatively correlated with insulin resistance in the body, increasing the synthesis of fatty acids in the body and hindering the synthesis of very low density lipoprotein. The output of triglycerides in the liver is reduced, and visceral and liver fats accumulate. A large number of studies have proved that insulin resistance is an important risk factor for NAFLD [30, 31]. The protective effect of vegetable foods on NAFLD can be mediated by the effect of beneficial ingredients on inflammatory markers (such as C-reactive protein and tumor necrosis factor α) [32, 33] due to the large amount of anti-inflammatory substances, such as vitamin C, vitamin E, and folic acid. These antioxidants can reduce the level of oxidative stress in the body and thus prevent the occurrence and development of NAFLD [34–36]. Fruit is rich in fructose. Excessive fructose can stimulate fat generation and inhibit mitochondrial fatty acid oxidation. High fructose intake will also increase the level of inflammation in the body [37], further elevating the level of oxidative stress in the body, leading to liver fibrosis, and increasing the risk of NAFLD [38–40].

Western dietary patterns were positively correlated with the risk of NAFLD, and our findings were consistent with current research results [25]. Western dietary patterns are characterized by high intake of red meat, processed meat, offal, white meat, fish, seafood, and alcoholic beverages. The intake of these foods may cause excess energy, which accumulates in the liver in the form of free fatty acids and promotes the synthesis of triacylglycerol. When the amount of excess triacylglycerol exceeds the transport capacity of the liver, it will gradually accumulate [41], which is the basic condition for the occurrence of fatty liver [42]. This phenomenon also increases the risk of obesity. In addition, excessive intake of red meat can increase the intake of saturated fatty acids and iron, which can reduce the oxidation of lipids and increase the synthesis of lipids and the accumulation of lipids in the liver. It can also promote insulin secretion to a certain extent, whereas maintaining a high level of insulin in the body for a long time can lead to β-cytotoxicity and functional failure, resulting in reduced insulin sensitivity and inducing insulin resistance [43–45]. Iron increases the level of oxidative stress in the body, thus increasing the risk of NAFLD [46]. Therefore, reducing the dietary intake of saturated fatty acids is an important measure for dietary prevention and treatment of NAFLD [47].

The cereal-potato dietary pattern is characterized by a high intake of whole grains and potatoes, among others. The cereal–potato dietary pattern was associated with a reduced risk of NAFLD (OR = 0.581, *p* < 0.05). First, high intake of whole grains promotes fat reduction [48, 49] and reduces the likelihood of obesity, which has been confirmed to be an important risk factor in NAFLD development [50]. Second, high intake of whole grains reduces oxidative stress and inflammatory markers [51], resulting in a decrease in liver enzymes and steatosis, thereby reducing the risk of NAFLD [52, 53]. Finally, whole grain foods are a good source of micronutrients such as copper, and NAFLD patients show a decrease in hepatic copper concentration, which has a positive effect on the reduction of the risk of NAFLD development.

The study has several limitations. First, meal frequency status was obtained from self-reports of survey respondents, making the data subject to some recall bias. Second, although as many factors as possible were corrected in the study, it may still be necessary to exclude the effects of other confounding factors. Third, this study is a cross-sectional survey study, but it is still not possible to determine causality based on our results. A large-scale, long-term longitudinal study may still be needed.

There are several strengths and limitations in this study. First, the residents were mostly from ethnic minorities in Gongcheng Yao Autonomous County, Guangxi, and the area has been the “hometown of longevity in China,” to the best of our knowledge, this was the first study investigating the relationships between different dietary patterns and the risk of NAFLD in this area. Second, the use of a validated semi-quantitative FFQ by a face-to-face interview ensured that the data we collected were accurate. Furthermore, for reliability, we had adjusted for potential known confounders in our analyses. However, there were several possible limitations. First, the lack of biomarker information on fibrosis (e.g., FIB-4 or NALFD fibrosis score) prevented us from assessing whether dietary patterns could affect fibrosis and from predicting patients’ long-term prognosis. Second, the present study was a cross-sectional study and could not determine the causal relationship between dietary patterns and the onset of NAFLD.

## Conclusion

The current study suggests that the Western dietary pattern is associated with increased risk of NAFLD, whereas the cereal–potato dietary pattern is associated with decreased risk of NAFLD. It is suggested that patients with NAFLD or those at high risk for its development should pay attention to controlling the intake of meat and sugary foods, increasing the intake of whole grain foods to improve the dietary structure and reduce the risk of NAFLD. Our findings may provide a reference for the preventive control of NAFLD. However, more studies examining longitudinal changes in NAFLD associated with dietary patterns specific are needed to clarify and build on the associations observed in the current study.

## Data Availability

The datasets used and/or analyzed during the current study are available from the corresponding author on reasonable request.

## Abbreviations

BMI: Body mass index
WC: Waist circumference
SBP: Systolic blood pressure
DBP: Diastolic blood pressure
FBG: Fasting blood glucose
HbA1c: Hemoglobin A1c
AST: Aspartate aminotransferase
ALT: Alanine aminotransferase
BP: Blood pressure
TC: Total cholesterol
HDL-C: High-density lipoprotein cholesterol
LDL-C: Low-density lipoprotein cholesterol
TG: Triglycerides
UA: Uric acid

## References

[1] Fitzpatrick E (2019). Understanding susceptibility and targeting treatment in non-alcoholic fatty liver disease in children; moving the fulcrum. Proc Nutr Soc.

[2] Younossi ZM, Koenig AB, Abdelatif D, Fazel Y, Henry L, Wymer M (2016). Global epidemiology of nonalcoholic fatty liver disease-Meta-analytic assessment of prevalence, incidence, and outcomes. Hepatology.

[3] Smits MM, Ioannou GN, Boyko EJ, Utzschneider KM (2013). Non-alcoholic fatty liver disease as an independent manifestation of the metabolic syndrome: results of a US national survey in three ethnic groups. J Gastroenterol Hepatol.

[4] Li Z, Xue J, Chen P, Chen L, Yan S, Liu L (2014). Prevalence of nonalcoholic fatty liver disease in mainland of China: a meta-analysis of published studies. J Gastroenterol Hepatol.

[5] Anstee QM, Targher G, Day CP (2013). Progression of NAFLD to diabetes mellitus, cardiovascular disease or cirrhosis. Nat Rev Gastroenterol Hepatol.

[6] Chalasani N, Younossi Z, Lavine JE, Diehl AM, Brunt EM, Cusi K, Charlton M, Sanyal AJ (2012). The diagnosis and management of non-alcoholic fatty liver disease: practice guideline by the american Gastroenterological Association, American Association for the study of Liver Diseases, and American College of Gastroenterology. Gastroenterology.

[7] Barshop NJ, Sirlin CB, Schwimmer JB, Lavine JE (2008). Review article: epidemiology, pathogenesis and potential treatments of paediatric non-alcoholic fatty liver disease. Aliment Pharmacol Ther.

[8] Nguyen V, George J (2015). Nonalcoholic fatty liver Disease Management: Dietary and Lifestyle modifications. Semin Liver Dis.

[9] Ristic-Medic D, Bajerska J, Vucic V (2022). Crosstalk between dietary patterns, obesity and nonalcoholic fatty liver disease. World J Gastroenterol.

[10] Zelber-Sagi S, Ratziu V, Oren R (2011). Nutrition and physical activity in NAFLD: an overview of the epidemiological evidence. World J Gastroenterol.

[11] Antonucci L, Porcu C, Iannucci G, Balsano C, Barbaro B (2017). Non-alcoholic fatty liver Disease and Nutritional Implications: special focus on copper. Nutrients.

[12] Gao X, Fan JG (2013). Diagnosis and management of non-alcoholic fatty liver disease and related metabolic disorders: consensus statement from the Study Group of Liver and Metabolism, Chinese Society of Endocrinology. J Diabetes.

[13] Williams R, Ashton K, Aspinall R, Bellis MA, Bosanquet J, Cramp ME, Day N, Dhawan A, Dillon J, Dyson J (2015). Implementation of the Lancet Standing Commission on Liver Disease in the UK. Lancet.

[14] Chalasani N, Younossi Z, Lavine JE, Diehl AM, Brunt EM, Cusi K, Charlton M, Sanyal AJ (2012). The diagnosis and management of non-alcoholic fatty liver disease: practice Guideline by the American Association for the study of Liver Diseases, American College of Gastroenterology, and the american Gastroenterological Association. Hepatology.

[15] Hu FB (2002). Dietary pattern analysis: a new direction in nutritional epidemiology. Curr Opin Lipidol.

[16] Wang J, Lin X, Bloomgarden ZT, Ning G (2020). The Jiangnan diet, a healthy diet pattern for chinese. J Diabetes.

[17] Chen C, Lu FC (2004). The guidelines for prevention and control of overweight and obesity in chinese adults. Biomed Environ Sci.

[18] World Health Organization (2012). Global physical activity questionnaire (Gpaq) Analysis Guide.

[19] Liu X, Wang X, Lin S, Song Q, Lao X, Yu IT (2015). Reproducibility and validity of a food frequency questionnaire for assessing Dietary Consumption via the Dietary Pattern Method in a Chinese Rural Population. PLoS ONE.

[20] Zhen S, Ma Y, Zhao Z, Yang X, Wen D (2018). Dietary pattern is associated with obesity in chinese children and adolescents: data from China Health and Nutrition Survey (CHNS). Nutr J.

[21] Nascimbeni F, Pais R, Bellentani S, Day CP, Ratziu V, Loria P, Lonardo A (2013). From NAFLD in clinical practice to answers from guidelines. J Hepatol.

[22] Ciardullo S, Oltolini A, Cannistraci R, Muraca E, Perseghin G (2022). Sex-related association of nonalcoholic fatty liver disease and liver fibrosis with body fat distribution in the general US population. Am J Clin Nutr.

[23] Vos MB, Colvin R, Belt P, Molleston JP, Murray KF, Rosenthal P, Schwimmer JB, Tonascia J, Unalp A, Lavine JE (2012). Correlation of vitamin E, uric acid, and diet composition with histologic features of pediatric NAFLD. J Pediatr Gastroenterol Nutr.

[24] Yang CQ, Shu L, Wang S, Wang JJ, Zhou Y, Xuan YJ, Wang SF (2015). Dietary patterns modulate the risk of non-alcoholic fatty liver disease in chinese adults. Nutrients.

[25] Liu X, Peng Y, Chen S, Sun Q (2018). An observational study on the association between major dietary patterns and non-alcoholic fatty liver disease in chinese adolescents. Med (Baltim).

[26] York LW, Puthalapattu S, Wu GY (2009). Nonalcoholic fatty liver disease and low-carbohydrate diets. Annu Rev Nutr.

[27] Bahrami A, Teymoori F, Eslamparast T, Sohrab G, Hejazi E, Poustchi H, Hekmatdoost A (2019). Legume intake and risk of nonalcoholic fatty liver disease. Indian J Gastroenterol.

[28] Rebello CJ, Greenway FL, Finley JW (2014). A review of the nutritional value of legumes and their effects on obesity and its related co-morbidities. Obes Rev.

[29] Schwingshackl L, Hoffmann G (2013). Long-term effects of low glycemic index/load vs. high glycemic index/load diets on parameters of obesity and obesity-associated risks: a systematic review and meta-analysis. Nutr Metab Cardiovasc Dis.

[30] Mouzaki M, Allard JP (2012). The role of nutrients in the development, progression, and treatment of nonalcoholic fatty liver disease. J Clin Gastroenterol.

[31] Barazzoni R, Deutz N, Biolo G, Bischoff S, Boirie Y, Cederholm T, Cuerda C, Delzenne N, Leon SM, Ljungqvist O (2017). Carbohydrates and insulin resistance in clinical nutrition: recommendations from the ESPEN expert group. Clin Nutr.

[32] Watzl B, Kulling SE, Moseneder J, Barth SW, Bub A (2005). A 4-wk intervention with high intake of carotenoid-rich vegetables and fruit reduces plasma C-reactive protein in healthy, nonsmoking men. Am J Clin Nutr.

[33] Hermsdorff HH, Zulet MA, Puchau B, Martinez JA (2010). Fruit and vegetable consumption and proinflammatory gene expression from peripheral blood mononuclear cells in young adults: a translational study. Nutr Metab (Lond).

[34] Harrison SA, Torgerson S, Hayashi P, Ward J, Schenker S (2003). Vitamin E and vitamin C treatment improves fibrosis in patients with nonalcoholic steatohepatitis. Am J Gastroenterol.

[35] Arendt BM, Allard JP (2011). Effect of atorvastatin, vitamin E and C on nonalcoholic fatty liver disease: is the combination required?. Am J Gastroenterol.

[36] Villaca CG, Pereira SE, Saboya CJ, Ramalho A (2008). Non-alcoholic fatty liver disease and its relationship with the nutritional status of vitamin A in individuals with class III obesity. Obes Surg.

[37] Rayssiguier Y, Gueux E, Nowacki W, Rock E, Mazur A (2006). High fructose consumption combined with low dietary magnesium intake may increase the incidence of the metabolic syndrome by inducing inflammation. Magnes Res.

[38] Toshimitsu K, Matsuura B, Ohkubo I, Niiya T, Furukawa S, Hiasa Y, Kawamura M, Ebihara K, Onji M (2007). Dietary habits and nutrient intake in non-alcoholic steatohepatitis. Nutrition.

[39] Softic S, Cohen DE, Kahn CR (2016). Role of Dietary Fructose and hepatic De Novo Lipogenesis in fatty liver disease. Dig Dis Sci.

[40] Abdelmalek MF, Suzuki A, Guy C, Unalp-Arida A, Colvin R, Johnson RJ, Diehl AM (2010). Increased fructose consumption is associated with fibrosis severity in patients with nonalcoholic fatty liver disease. Hepatology.

[41] Holterman AX, Guzman G, Fantuzzi G, Wang H, Aigner K, Browne A, Holterman M (2013). Nonalcoholic fatty liver disease in severely obese adolescent and adult patients. Obes (Silver Spring).

[42] Donnelly KL, Smith CI, Schwarzenberg SJ, Jessurun J, Boldt MD, Parks EJ (2005). Sources of fatty acids stored in liver and secreted via lipoproteins in patients with nonalcoholic fatty liver disease. J Clin Invest.

[43] Chung S, Parks JS (2016). Dietary cholesterol effects on adipose tissue inflammation. Curr Opin Lipidol.

[44] Kim Y, Keogh J, Clifton P (2015). A review of potential metabolic etiologies of the observed association between red meat consumption and development of type 2 diabetes mellitus. Metabolism.

[45] Choi Y, Lee JE, Chang Y, Kim MK, Sung E, Shin H, Ryu S (2016). Dietary sodium and potassium intake in relation to non-alcoholic fatty liver disease. Br J Nutr.

[46] Alla V, Bonkovsky HL (2005). Iron in nonhemochromatotic liver disorders. Semin Liver Dis.

[47] Bhatt SP, Misra A, Nigam P (2019). Nutrition and physical activity in asian Indians with non-alcoholic fatty liver: a case control study. Diabetes Metab Syndr.

[48] Katcher HI, Legro RS, Kunselman AR, Gillies PJ, Demers LM, Bagshaw DM, Kris-Etherton PM (2008). The effects of a whole grain-enriched hypocaloric diet on cardiovascular disease risk factors in men and women with metabolic syndrome. Am J Clin Nutr.

[49] Kristensen M, Toubro S, Jensen MG, Ross AB, Riboldi G, Petronio M, Bugel S, Tetens I, Astrup A (2012). Whole grain compared with refined wheat decreases the percentage of body fat following a 12-week, energy-restricted dietary intervention in postmenopausal women. J Nutr.

[50] Zeng J, Yang RX, Sun C, Pan Q, Zhang RN, Chen GY, Hu Y, Fan JG (2020). Prevalence, clinical characteristics, risk factors, and indicators for lean chinese adults with nonalcoholic fatty liver disease. World J Gastroenterol.

[51] Hajihashemi P, Azadbakht L, Hashemipor M, Kelishadi R, Esmaillzadeh A (2014). Whole-grain intake favorably affects markers of systemic inflammation in obese children: a randomized controlled crossover clinical trial. Mol Nutr Food Res.

[52] Sun T, Deng Y, Geng X, Fang Q, Li X, Chen L, Zhan M, Li D, Zhu K, Li H (2022). Plasma Alkylresorcinol Metabolite, a biomarker for whole-grain intake, is inversely Associated with risk of nonalcoholic fatty liver disease in a case-control study of chinese adults. J Nutr.

[53] Dorosti M, Jafary HA, Bakhshimoghaddam F, Alizadeh M (2020). Whole-grain consumption and its effects on hepatic steatosis and liver enzymes in patients with non-alcoholic fatty liver disease: a randomised controlled clinical trial. Br J Nutr.

